# Nuclear Reprogramming: Kinetics of Cell Cycle and Metabolic Progression as Determinants of Success

**DOI:** 10.1371/journal.pone.0035322

**Published:** 2012-04-18

**Authors:** Sebastian Thomas Balbach, Telma Cristina Esteves, Franchesca Dawn Houghton, Marcin Siatkowski, Martin Johannes Pfeiffer, Chizuko Tsurumi, Benoit Kanzler, Georg Fuellen, Michele Boiani

**Affiliations:** 1 Max Planck Institute for Molecular Biomedicine, Münster, Germany; 2 Faculty of Medicine, University of Southampton, Southampton General Hospital, Southampton, United Kingdom; 3 German Center for Neurodegenerative Disorders, DZNE, Rostock, Germany; 4 Institute for Biostatistics and Informatics in Medicine and Ageing Research, University of Rostock, Rostock, Germany; 5 Department of Radiation Oncology, University Hospital Freiburg, Freiburg, Germany; 6 Max Planck Institute of Immunobiology and Epigenetics, Freiburg, Germany; University of Connecticut, United States of America

## Abstract

Establishment of totipotency after somatic cell nuclear transfer (NT) requires not only reprogramming of gene expression, but also conversion of the cell cycle from quiescence to the precisely timed sequence of embryonic cleavage. Inadequate adaptation of the somatic nucleus to the embryonic cell cycle regime may lay the foundation for NT embryo failure and their reported lower cell counts. We combined bright field and fluorescence imaging of histone H_2b_-GFP expressing mouse embryos, to record cell divisions up to the blastocyst stage. This allowed us to quantitatively analyze cleavage kinetics of cloned embryos and revealed an extended and inconstant duration of the second and third cell cycles compared to fertilized controls generated by intracytoplasmic sperm injection (ICSI). Compared to fertilized embryos, slow and fast cleaving NT embryos presented similar rates of errors in M phase, but were considerably less tolerant to mitotic errors and underwent cleavage arrest. Although NT embryos vary substantially in their speed of cell cycle progression, transcriptome analysis did not detect systematic differences between fast and slow NT embryos. Profiling of amino acid turnover during pre-implantation development revealed that NT embryos consume lower amounts of amino acids, in particular arginine, than fertilized embryos until morula stage. An increased arginine supplementation enhanced development to blastocyst and increased embryo cell numbers. We conclude that a cell cycle delay, which is independent of pluripotency marker reactivation, and metabolic restraints reduce cell counts of NT embryos and impede their development.

## Introduction

Somatic cell nuclear transfer (NT) into mouse oocytes efficiently yields pluripotent stem cells [1], which are functionally and transcriptionally indistinguishable from those derived from fertilized embryos [2], [3], [4]. However, pregnancies from NT mouse embryos are rare, and cloned offspring are exceptional [5]. To explain the discrepancy between stem cell and pregnancy rates, it has been proposed that paternal alleles of genes involved in trophectodermal differentiation and placenta formation may be refractory to reprogramming [6]. Critical embryonic genes must be activated in less than 24 hours (embryonic genome activation) in order for the somatic nucleus to support cleavage when maternal transcripts disappear [7]. In contrast, transcription factor-induced direct cell reprogramming tolerates extended latency [8]. Since embryonic development proceeds as precisely timed sequence of events [9], [10], [11] and is characterized by rapid successions of DNA replication and mitotic divisions lacking notable G_1_ or G_2_ phases, the long cell cycle of differentiated cells [12] must therefore be reprogrammed after NT to comply with the embryonic program.

There are several reports indicating that the cell cycle of NT mouse embryos may be paralyzed by their somatic heritage [5], [13]. As a consequence, NT blastocysts suffer low cell counts. These may be explained by three scenarios: a) a cloned embryo passes through the cell cycle with reduced speed, and/or b) some of its cells undergo apoptosis, and/or c) exit the cell cycle into a quiescent state, caused by metabolic restraints, checkpoint activation or incomplete reprogramming of essential embryonic genes. While cells of cloned embryos undergo apoptosis as infrequently as controls (scenario b) [5], the reported aberrant gene expression patterns at the first two cleavage stages [7] could halt NT blastomeres (scenario c). Determination of cell cycle progression (scenario a) relies in large part on live embryo cinematography [14], whose necessary light exposure is highly toxic to cloned embryos. However, if the toxicity to cells of live cell cinematography during cell cycle analysis can be minimized, it has the potential to reveal otherwise undetectable embryo features. Overall quantification of cell cleavage dynamics has more technical difficulties than gene expression profiling.

In studies of NT embryos, there is a tendency to consider only those embryos that have reached predefined key stages (e.g., morula or blastocyst), that is, after completion of the bulk of reprogramming. In this retrospective approach it is impossible to separate successful and non-successful embryos before analysis. Non-detrimental prediction of developmental success is therefore essential to elucidate mechanisms of reprogramming. Progression of human in vitro fertilized (IVF) embryos to the blastocyst stage can be predicted by determining cleavage timings of the two-cell blastomeres [15]. In cloned mouse embryos, the timing of the first cleavage may be less informative about reprogramming because entry into M phases of the first two cleavage divisions is regulated independently of the (reprogrammed) nucleus by activation of M phase-promoting factor, MPF [16]. However, the timing of completion of the third cell cycle may be a critically important parameter to predict successful reprogramming.

Time-lapse cinematography also grants real-time insight into the behavior of cloned embryos in different culture environments. For cloned but not for fertilized embryos, the culture medium has large impact on blastocyst formation [17] and on the distribution of the pluripotent stem cell marker Oct4 [18], suggesting distinct culture media requirements [19]. Culture media differ in composition of carbohydrates, salts, vitamins and, in particular, amino acids. Amino acids serve as building blocks of proteins and as energy sources, and regulate pH and osmolarity [20] and have been assigned intra-cellular signaling functions during embryo pre-implantation development [21]. Amino acid turnover is a marker of early mammalian embryo viability [22], [23].

We have devised a transgenic mouse tool, H_2b_-GFP, to analyze the cell cycle progression of NT embryos up to the 16-cell stage by combining bright field and fluorescence live-cell imaging. We then correlated cell cycle kinetics with blastocyst formation and gene expression. Cloned embryos showed an extended duration of the second and third cell cycles compared to fertilized counterparts. Although the cell cycle pace of cloned embryos predicted blastocyst formation, transcriptome analysis detected marginal differences between fast and slow NT embryos. Metabolic profiling revealed that NT embryos consume lower amounts of amino acids, in particular arginine, than fertilized controls until morula stage. Culture medium supplementation with arginine facilitated blastocyst formation of cloned embryos. We conclude that cell cycle progression and pluripotency marker reactivation are independent features of oocyte-mediated reprogramming.

## Results

### Transgene enables viable time-lapse cinematography of cloned mouse embryos

**Figure 1:**
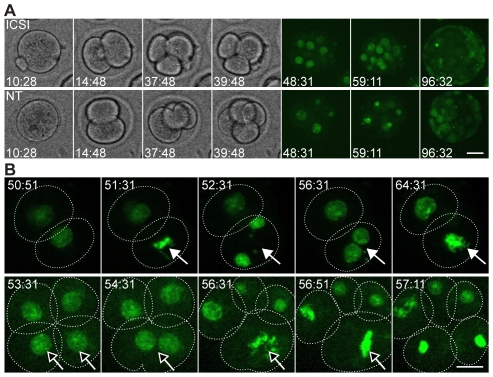
Time-lapse cinematography of ICSI and NT mouse embryos. A) Until 48 hours post activation (hpa), bright-field images were captured every 20 minutes. From 48 until 96 hpa, confocal optical sections of *H2b-GFP* expressing embryos were captured every 20 minutes. Time-lapse movies were evaluated to obtain the timing of cleavages. B) Cell division aberrancies such as failing cytokinesis (top panel; filled arrows) or cell fusions (bottom panel; empty arrows) were detected in both NT and ICSI embryos. Dotted lines indicate cell membranes. Scale bar, 20 µm.

While direct cell reprogramming induced by transcription factors tolerates different cell division rates [8], an NT embryo that fails to adapt to the embryonic cleavage regime may be selected against. Therefore, a systematic dissection of the first cell cycles of cloned mouse embryos could unveil important mechanisms related to somatic reprogramming and cell cycle regulation. However, cell cycle analysis of embryos cloned by nuclear transfer (NT) is difficult to carry out because their high vulnerability to light hampers time-lapse cinematography. For example, using protocols for time-lapse cinematography considered safe for mouse fertilized embryos [14], there was two-cell stage arrest in NT embryos while ICSI embryos formed blastocysts (movie S1). We devised a combined bright field and fluorescence time-lapse cinematography protocol that improved survival of NT embryos. We used an interference bandpass filter (580/10 nm) for bright field to exclude harmful wavelengths, and generated a mouse line ubiquitously and constitutively expressing a histone H_2b_-GFP transgene. With these tools we determined cell cycle lengths of the first four cell cycles of mouse embryos cloned from cumulus cells and control embryos fertilized by intra-cytoplasmic sperm injection (ICSI), during culture in α-MEM (Figure 1A; movies S2, S3). For each cell of every embryo, the time between consecutive cleavages was determined and development to the blastocyst stage was tracked. We then analyzed correlation between cell cycle length and development to the blastocyst stage. Additionally, we recorded gross M phase aberrancies (multi- or unipolar spindle, chromatid nondisjunction, cell fusion, cytokinesis failure; Figure 1B; movies S4, S5). Imaged fertilized embryos developed equally well as embryos in the incubator (table S1). Although imaging conditions were very mild, rates of development of cloned embryos were not as high as for non-imaged controls (13.4% versus 33.9%; table S1). However, since fertilized control embryos were always imaged in parallel with cloned embryos in the same session, conclusions drawn from comparative analysis are considered as valid.

### Dramatic differences in cleavage timing of cloned embryos show low correlation to post-implantation development

**Figure 2:**
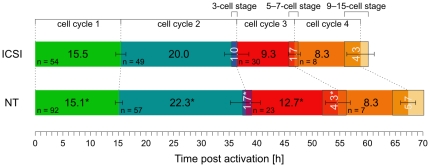
Cell cycle duration of ICSI and NT mouse embryos in hours, starting from activation. Green, cell cycle 1 (one-cell stage); blue, cell cycle 2 (two-cell stage); red, cell cycle 3 (four-cell stage); orange, cell cycle 4 (eight-cell stage). Boxed, inter-stages (three-cell stage, five- to seven-cell stage, nine- to fifteen-cell stage). Centred black numbers indicate median length of the entire cleavage stage; white numbers indicate the inter-stage length only. Error bars, median average deviation of entire cleavage stage length; *n*, number of embryos at respective stage. *, significantly different from same cell cycle in ICSI (Wilcoxon rank sum test, *p*<0.01).

We observed that the length of the first cell cycle was slightly but significantly shorter in cloned compared with fertilized embryos, possibly due to the different activation method. The second and in particular the third cell cycles were substantially longer in NT embryos (plus 2.3 hours and plus 3.4 hours, respectively, Wilcoxon rank sum test, *p* = 5.31⋅10^−4^ and *p* = 3.16⋅10^−5^, respectively; Figure 2). This difference is not due to cell cycle speed variability between different strains of mice, as previously reported [24], [25], [26], as in our study cloned and fertilized control embryos shared the same genetic background. Interestingly, the fourth cell cycle was not different between cloned and fertilized embryos (*p* = 0.953). It is not surprising that we found only a minor difference in first cell cycle of cloned and fertilized embryos, as this cleavage is determined nucleus-independently by maternal factors [16], which should be equally present in the cytoplasm of both types of embryos. In the mouse, the embryonic genome is activated at the late two-cell stage (major embryonic genome activation), consistent with the longer second cell cycle [27]. The dramatic slowdown of cloned embryos precisely coinciding with embryonic genome activation suggests delayed re-expression of essential cell cycle genes from quiescent somatic donor cells. While maternal proteins may still be sufficient for transit through two-cell stage – albeit with limited speed –, cloned embryos may be forced to extend the four-cell stage to wait for replenishment of cell cycle molecules. Reduction of Zscan4 in mouse embryos leads to a similar phenotype [28]. If the cloned embryo fails to re-activate these essential genes, its cells arrest, resembling the observed two-cell block of Brg1-depleted mouse oocytes [29] or when preventing protein synthesis [30]. This may well explain the high losses after NT at this developmental stage.

Cleavages were less synchronous for NT than for ICSI embryos: The median duration of the three-cell stage was 1.7 hours for NT and 1.0 hour for ICSI embryos (Wilcoxon rank sum test, *p* = 4.97⋅10^−3^), and the five- to seven-cell stage lasted 4.3 hours for NT and 1.7 hours for ICSI embryos (Wilcoxon rank sum test, *p* = 1.10⋅10^−4^; Figure 2). The variability of cell division speed, between embryos (error bars in Figure 2) but also between individual blastomeres of one cloned embryo, is much higher than that of ICSI embryos and suggests some degree of stochasticity in reprogramming of (cell cycle) genes. Once the necessary genes are re-activated however, NT embryos show the same cleavage pace as fertilized embryos, which would explain why the duration of the eight-cell stage is identical in cloned and fertilized embryos.

**Figure 3:**
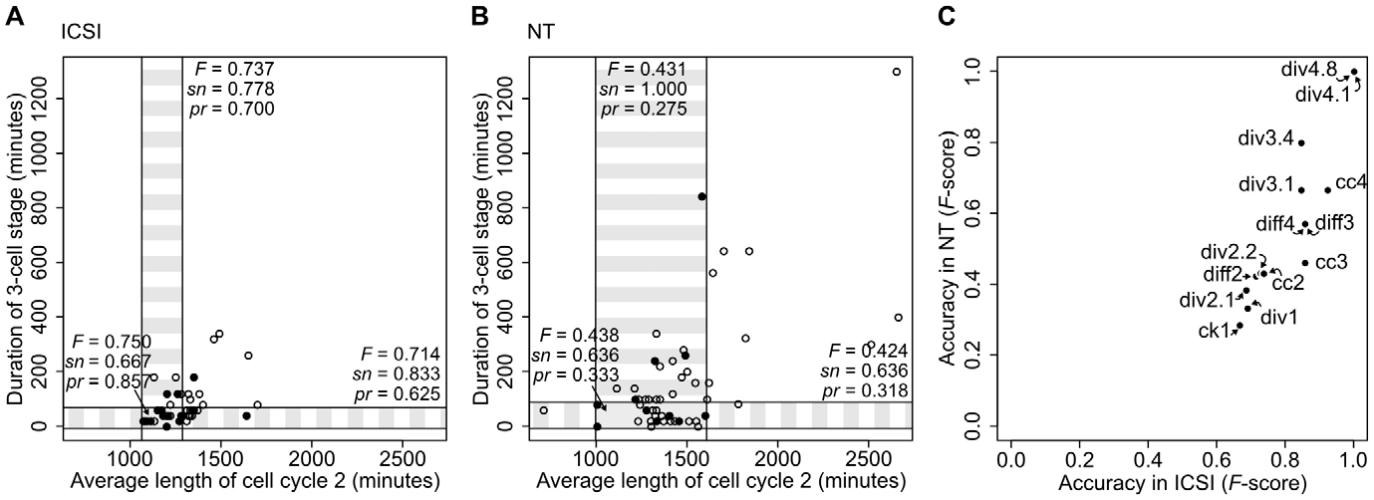
Accuracy of predicting developmental success from cleavage timings. A,B) Duration of three-cell stage plotted against the average length of cell cycle 2 of single embryos for ICSI and NT, respectively. Empty dots, embryos that did not develop to blastocyst; filled dots, embryos that developed to blastocyst. The window of highest prediction accuracy is highlighted, and F-score, 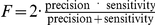, a combined measure of accuracy, sensitivity (*sn*) and precision (*pr*) of the predictions are indicated. The variability of cleavage timings is much higher between NT embryos than between ICSI embryos, therefore the time window for prediction are larger in NT. C) Accuracy of predicting blastocyst formation by particular measures of cell cycles in cloned (NT) and fertilized (ICSI) embryos up to the 16-cell stage (F-score). cc2, cc3, cc4, average length of cell cycles 2, 3, 4, respectively; ck1, duration of 1^st^ cytokinesis; diff2, diff3, diff4, length of inter-stages, i.e. 3-cell, 5- to 7-cell, 9- to 15-cell stages, respectively; div1, div2.1, div2.2, div3.1, div3.4, div4.1, div4.8, time of division to 2-, 3-, 4-, 5-, 8-, 9-, 16-cell stages, respectively. Until 4-cell stage, there are no measured variables predicting blastocyst formation of NT embryos with *F*>0.45.

It has been reported that progression of human embryos to the blastocyst stage can be predicted with high accuracy before the stage of embryonic genome activation, by measuring the time between consecutive divisions and the duration of the first cytokinesis [15]. However, after analysis of these parameters (Figure 3) we were unable to predict developmental success of mouse NT embryos from cleavage speed with the accuracy reported for human embryos [15]. For ICSI embryos, the length of the first cell cycle already predicted development to the blastocyst stage with an accuracy of 66.7% (Figure 3), but for NT embryos the predictive value of cleavage timings were lower. In fact, for cloned embryos only parameters later than four-cell stage predicted blastocyst development with 66.7% or more (Figure 3); combination of earlier parameters did not increase accuracy of prediction to over 48.9% either (figures S1, S2). ICSI embryos were consistent in their cleavage pace, that is, a blastomere that cleaved early was likely to cleave early in the next cell cycle, too (first versus second cell cycle, Pearson correlation coefficient *r* ^2^ = 0.281, second versus third, *r* ^2^ = 0.381, third versus fourth, *r* ^2^ = 0.474; figure S3). NT embryos only maintained their cleavage speed after the eight-cell stage (third versus fourth cell cycle, *r* ^2^ = 0.439). Their second and third cell cycles were negatively correlated (*r* ^2^ = 0.476), indicating that cloned embryos benefit from a longer two-cell stage, leading to faster development afterwards. However, in both ICSI and NT embryos cleavage was non-cell autonomous, that is, if one cell divided, the sister cell was likely to divide as well (Fisher's exact test, *p*<2.2⋅10^−16^). Also, the duration of the cell cycle for a particular cell and its sister cell always correlated (ICSI, *r* ^2^ = 0.948, NT, *r* ^2^ = 0.815; figure S4).

Since the above results suggest that cleavage timings reflect embryo quality for fertilized embryos (as reported before [31] and measured here by developmental rates), but less so for cloned embryos, we analyzed post-blastocyst development. We classified cloned embryos as *fast* or *slow* based on their timing to divide to three-cell stage at 35 to 41 hours post activation, and assessed blastocyst formation, embryonic stem cell (ESC) derivation and fetal formation (Table 1). *Fast* NT embryos were more often successful at forming blastocysts, but fetal formation was not different between *fast* and *slow*. This suggests that genes determining cell cycle speed in cloned embryos at early developmental stages are reprogrammed independently from genes required for successful post-implantation development. Derivation of ESCs was almost twice as efficient from slow as from fast-dividing NT embryos, with difference showing marginal significance (Table 1). Pluripotency-related genes may therefore be more efficiently reprogrammed in slow-dividing cloned embryos.

**Table 1:** Cleavage pace predicts blastocyst formation of cloned embryos.

Embryo type	% blastocysts (*n* four-cells)	% ESC lines (*n* outgrowths; *n* blastocysts plated)	% fetuses E10.5 (*n* implantations; *n* four-cells transferred)
NT fast	48.9 (264)	8.4 (46; 107)	2.1 (54; 145)
NT slow	27.8 (299)	17.4 (31; 69)	1.1 (67; 178)
*p*	2.6·10^−7^	0.095	0.660
ICSI	41.4 (428)	15.8 (52; 77)	32.8 (88; 180)

Embryos were scored *fast* or *slow* according to the time spent until the three-cell stage was reached. Then blastocyst formation was recorded, and ESCs were derived (data pooled from 4 experiments), or fetal rates were determined at E10.5 after transfer in utero 12 hours after scoring (data pooled from 2 experiments). The *p*-value of Fisher's exact test shows that the difference in blastocyst formation of *fast* and *slow* is significant, difference in ESC formation efficiency is of borderline significance, and difference in fetal formation is not significant. Data were pooled from five independent NT experiments. Development data from fertilized control embryos (ICSI) are shown in bottom row. Note that fertilized embryos were not sorted into fast and slow, and therefore frequencies relate to the two-cell stage. Derived ESCs were pluripotent regardless of their origin (fast, slow) as demonstrated by in vitro differentiation into derivatives of the three germ layers (figure S5, movies S6, S7, S8) and by teratoma formation (data not shown).

### Small differences in gene expression of fast- and slow-dividing NT embryos

**Figure 4:**
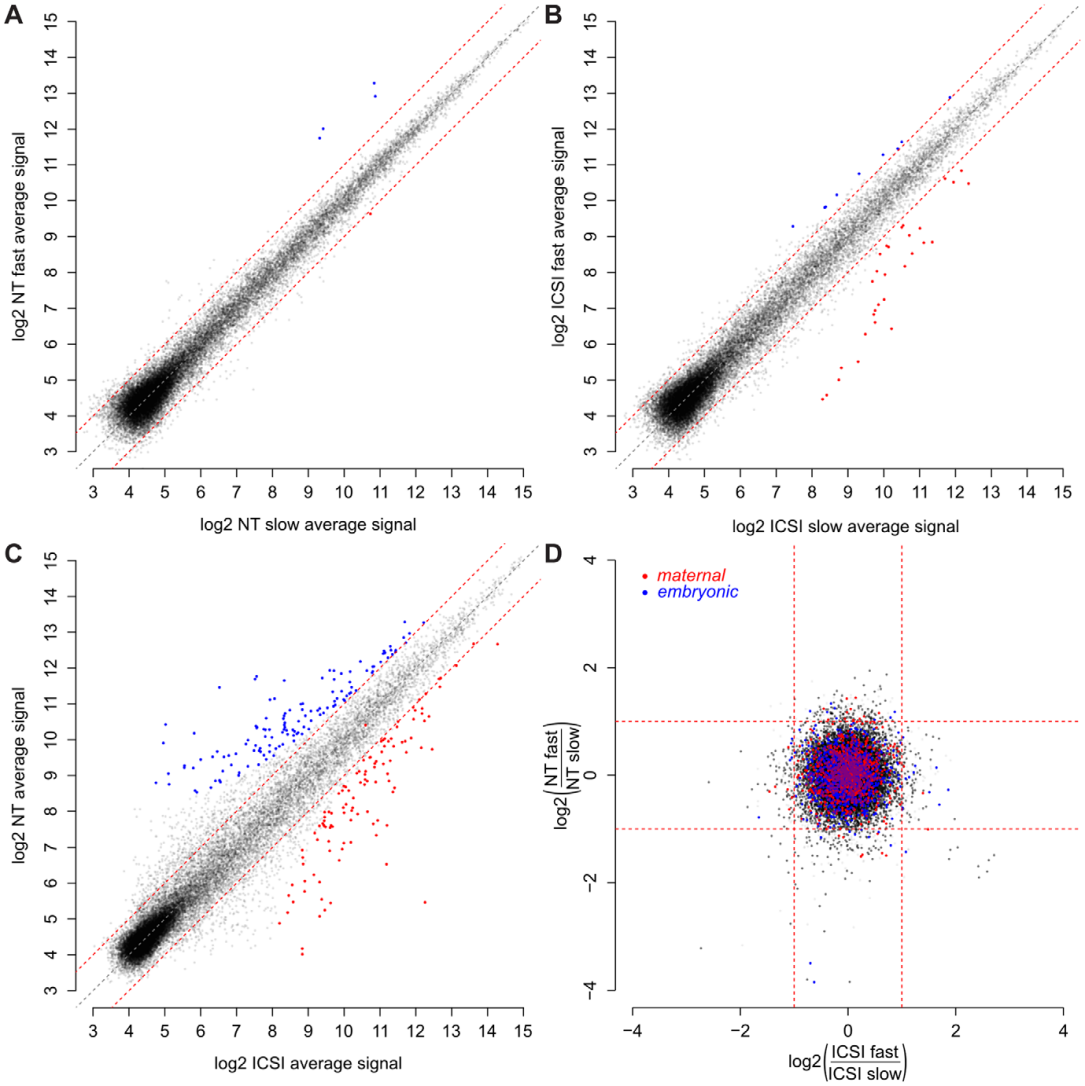
Scatter plot of gene expression levels. A) NT *fast* and NT *slow* largely overlap (5 genes differently expressed, red/blue dots), B) ICSI *fast* and ICSI *slow* also largely overlap (34 genes differently expressed, red/blue dots), C) NT and ICSI show great differences in gene expression (218 genes differently expressed, red/blue dots). D) Fold-change of gene expression in ICSI *fast* versus ICSI *slow* and NT *fast* versus NT *slow*. Red dots, oocyte/1-cell-specific (*maternal*) transcripts; blue dots, 4-cell/blastocyst-specific transcripts (*embryonic*); black dots, transcripts not different between maternal and embryonic stages; grey dots, transcripts not represented in downloaded data sets. Embryonic transcripts are over-represented in *fast* ICSI embryos.

The observed differences in developmental potential of fast- versus slow-developing cloned embryos suggest that cell cycle speed either affects or reflects reprogramming efficiency. To explore these possibilities, we classified NT and ICSI embryos in three groups based on their timing to divide to three-cell stage at 35 and 41 hours post activation: *fast*, *intermediate* or *slow*. Using hybridization to Illumina whole-genome expression beadchips, we compared the gene expression patterns of *fast* and *slow* embryos when these had reached the eight-cell stage. We chose to let embryos cleave to eight-cell stage in order to exclude *slow* embryos that would not have divided. Differences of NT *fast* and *slow* embryos were only marginal (Figure 4A), and so were differences between ICSI *fast* and *slow* embryos (Figure 4B), although our microarray analysis detected dramatic differences between NT and ICSI eight-cell embryos (Figure 4C). *Fast* NT embryos expressed higher levels of *Hist1h2af*, *Hist1h2an*, *Hist1h2ap*, *Hist2h2ac*, and lower levels of *Ate1*. The former are essential nucleosomal core proteins whose expression is cell cycle dependent [32], and whose appearance in this study is likely owed to the different cell cycle stage of the two groups of embryos at the same collection time point. Ate1 mediates posttranslational protein arginylation [33], required for regulated degradation of specific proteins [34]. This suggests that there are no systematic differences in reprogramming between fast and slow developing cloned embryos, neither of cell cycle nor of pluripotency genes. Longer exposure of the somatic genome to maternal reprogramming factors in slow cleaving cloned embryos may facilitate expression of pluripotency-related genes at later stages and explain the more efficient derivation of ESCs from slow-developing NT embryos.

We used published data sets [35], [36], [37] to select genes specific to oocyte/one-cell stage – termed *maternal* – or specific to four-cell/blastocyst stage – termed *embryonic* – and checked for overrepresentation of *maternal* and *embryonic* genes in *slow* and *fast* embryos. Although differences of global gene expression between *fast* and *slow* embryos were minute, we found overrepresentation of *maternal* genes in *slow* ICSI embryos and of *embryonic* genes in *fast* ICSI embryos (Figure 4D, hypergeometric distribution, *p* = 0.053 and *p* = 2.7·10^−5^, respectively). This difference points to delayed degradation or increased stability of maternal transcripts in slow developing embryos and to more efficient activation of embryonic genes in fast developing embryos. Interestingly, *fast* and *slow* NT embryos show much fewer differences, pointing to non-systematic variance of gene expression and to a stochastic component of gene expression reprogramming.

Many genes underexpressed in NT compared to ICSI embryos are *embryonic* genes (e.g., *Cdx2*, *Dppa5a*, *Foxa1*, *Tead4*, *Zfp239*, *Zfp644*; *p* = 3.0·10^−10^). This suggests delayed activation of embryonic gene expression until eight-cell stage in cloned embryos. Interestingly, among these genes there was overrepresentation (*p*<0.0005) of genes whose mutations (loss-of-function) cause abnormal spongiotrophoblast layer morphology (MP:0004255), increased placenta weight (MP:0004920) or absent trophoblast giant cells (MP:0001714), according to a search in the mouse genome database [38]. This analysis suggests that NT post-implantation failure may already be anticipated at the eight-cell stage.

### NT cell cycle delay is not due to DNA damage, but NT embryos are endowed with lower tolerance to cell division failure

Cloned as well as ICSI-fertilized embryos may be forced to pause the cell cycle in order to repair DNA damage [39] caused by micromanipulation techniques [40], [41]. We therefore sought to test if the cell cycle delay observed in cloned embryos was due to DNA double-strand breaks, which would be expected if mechanical damage had occurred. We used an antibody against phosphorylated histone H2A.X (γH2A.X), a marker for DNA double-strand breaks [42], to compare DNA damage in cloned and fertilized embryos at the four- and eight-cell stage. ICSI-fertilized four-cell stage embryos showed slightly higher levels of γH2A.X than cloned embryos (integrated signal intensity; *t* test, *p* = 1.4·10^−3^). This suggests that the observed cell cycle delay of NT embryos was not caused by higher DNA damage, under the experimental conditions of this study.

We have previously reported that cloned embryos present less aneuploidy than embryos fertilized by ICSI at the four-cell stage [40], in particular trisomy. However, after derivation of ESCs this relation had inverted [40]. It should be noted that chromosome behavior at mitosis may be contingent on the genotype of the cloned embryo [43]. In this context, we analyzed our time-lapse records for M phase errors leading to aneuploid daughter cells in the third cleavage division, and found that NT and ICSI embryos had similar rates of mitotic error (NT, 9 in 76; ICSI, 3 in 33; Fisher's exact test, *p* = 1). This argues that the cell cycle delay of cloned embryos is not caused by aneuploidy. Nevertheless, the longer the cell cycle, the more likely that M phase was aberrant, for both NT and control embryos (logistic regression coefficient significantly different from zero, *p* = 9.2·10^−4^ and *p* = 0.017, respectively). Interestingly, we did not observe any NT embryo developing to blastocyst stage after error in mitosis of a four-cell stage blastomere (*n* = 54), in contrast to ICSI embryos, which went to blastocyst in spite of such event (*n* = 23, Fisher's exact test, *p* = 1; table S2). Low frequencies of cell division aberrancies in early cloned embryos indicate that they acquire chromosomal aberrancies later in development, for example, after ESCs have been derived. Our results suggest that checkpoints are not (yet) reprogrammed in NT embryos at the four-/eight-cell division and therefore still halt cell cycle upon activation as reported for somatic cells [44] whereas fertilized embryos tolerate [45], [46] or even correct [47] aneuploid cells. This may provide a mechanism to explain low aneuploidy of early cloned embryos [40].

### Distinct amino acid metabolic profiles of cloned and fertilized embryos

Cloned embryos have culture media requirements different from fertilized embryos [17], [48]. Therefore, metabolic reasons may be at the root of the slow phenotype of cloned embryos. Amino acid turnover is a potential marker of early mammalian embryo viability [22], [23], [49]. We therefore analyzed the turnover (appearance and disappearance) of 18 amino acids in the spent culture media of NT and ICSI embryos at different stages of pre-implantation development (0–24 hpa, i.e. one-/two-cell; 24–48 hpa, two-/four-cell; 48–72 hpa, four-cell/morula; 72–96 hpa, morula/blastocyst stage). Total turnover increased with pre-implantation development, as also reported for porcine embryos [50].

**Figure 5:**
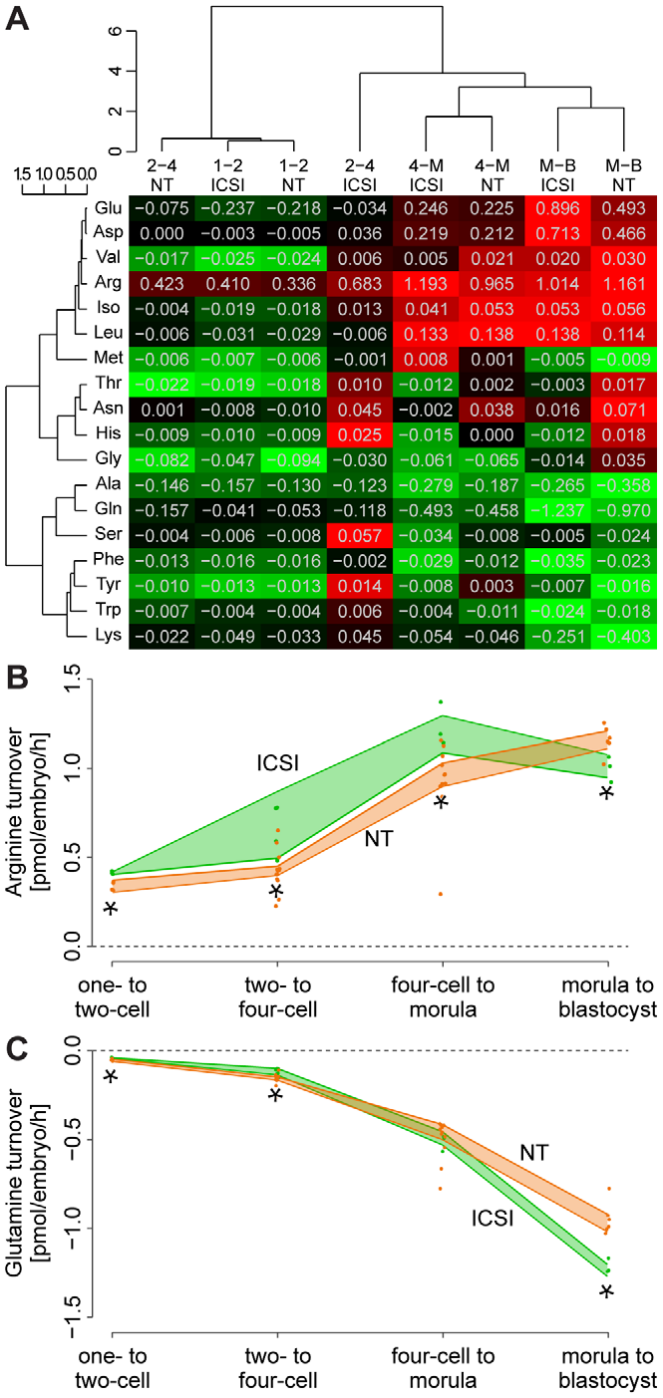
Analysis of amino acids in culture media of NT and ICSI pre-implantation embryos. A) Amino acid turnover profile of NT and ICSI embryos at 0–24, 24–48, 48–72 and 72–96 hours post activation, hpa, respective one-/two-cell (1–2), two-/four-cell (2–4), four-cell/morula (4–M) and morula/blastocyst (M–B) stages and unsupervised hierarchical clustering. Red, consumption; green, release into the culture medium; black, low turnover. At two-/four-cell stage, NT embryos cluster with earlier stages while ICSI cluster with later developmental stages, suggesting that NT embryos undergo the amino acid metabolic switch later than ICSI. Cell values indicate amino acid depletion/appearance in pmol·embryo^−1^·h^−1^. B) Arginine and C) glutamine turnover profile of ICSI and NT embryos over time. Arginine is depleted from the medium while glutamine is released; *, turnover significantly different between NT and ICSI (*t* test, *p*<0.05). Consumption of arginine by NT embryos is lower than by controls until morula stage.

Cloned and fertilized embryos had distinct amino acid turnover signatures (Figure 5A). For the first 24 hours of development, cloned embryos were highly similar to fertilized embryos (Pearson correlation coefficient *r* ^2^ = 0.863). At the two-/four-cell stage, cloned embryos lagged behind fertilized embryos, that is, they clustered with one-/two-cell stage embryos (*r* ^2^ = 0.805) but not with fertilized counterparts (*r* ^2^ = 0.022) while the latter were more similar to four-cell/morula stage. At four-cell/morula and at morula/blastocyst stage, the two embryo types were again similar in their amino acid turnover signature (*r* ^2^ = 0.630 and *r* ^2^ = 0.638). In summary, there was a clear metabolic switch from oocytic to embryonic amino acid turnover profile between the two- and the four-cell stage, which NT embryos implement about 24 hours later than fertilized controls (Figure 5A).

We also observed that total amino acid turnover was generally lower in cloned than in fertilized embryos. In particular, cloned embryos consume less arginine until the morula stage (Figure 5B), and less aspartate, glutamine (Figure 5C) and glycine until the four-cell stage. Interestingly, in mouse blastocysts, arginine is the amino acid most consumed in the inner cell mass (ICM) [51], an observation that implicates high arginine-dependent nitric oxide (NO) production [51]. High NO production may enforce the quiescent metabolic state of ICM because NO signaling lowers O_2_ consumption via interaction with cytochrome c oxidase in mitochondria [52]. Although the impact of NO on reprogramming has not been assessed directly, it has been reported that NO signaling induces *Oct4* expression in the hematopoietic system [53] and has impact on epigenetic modification [54]. Furthermore, arginine's metabolic product ornithine has been implicated in cell proliferation, differentiation and repair [55], [56]. Interestingly, in our study, the relation of arginine consumption became inverted at the morula/blastocyst stage, with cloned embryos having higher consumption than fertilized controls (Figure 5B). Because trophectoderm and ICM have different turnover of arginine [51], the erroneous cell lineage allocation of cloned blastocysts compared to fertilized controls [57] may contribute to the arginine metabolism phenotype.

### Arginine supplementation improves blastocyst formation

The differences in arginine metabolism of cloned embryos prompted us to directly probe arginine's influence on cloned embryo cell cycle and development. We cultured NT embryos with double the amount of arginine usually present in α-MEM medium (0.6 mmol/L). Indeed, with twofold arginine blastocyst formation was enhanced (1× arginine, 48/155; 2× arginine, 70/155; Fisher's exact test, *p* = 0.014) and cell counts of blastocysts were increased (average number of cells per blastocyst 29.7 versus 39.2, Wilcoxon rank sum test, *p* = 0.088). This effect was specific for arginine, as adding the same amount of glutamine did not facilitate blastocyst formation (data not shown). The effect was also specific for cloned embryos, as blastocyst formation of fertilized embryos did not change (data not shown). We also measured cell cycle progression of both cloned and fertilized embryos with the double amount of arginine using live cell imaging; however, we did not observe an acceleration of development.

Possible reasons for enhanced cloned embryo development include 1) a reduced selective pressure on cloned embryos by increased supply of rate-limiting arginine in the culture medium, and 2) a positive effect of higher arginine supply on reprogramming, for example, through NO signaling. The first explanation seems unlikely, as amino acid concentration in the culture medium exceeds demands (estimated from uptake) by at least 6.7 orders of magnitude. We therefore challenged the second hypothesis by adding an NO donating drug (DETA/NO, 10 µM), however, cloned embryos did not benefit (DETA/NO, 28/80 blastocysts; control, 33/81; Fisher's exact test, *p* = 0.517). We conclude that the beneficial effect of arginine to cloned embryo pre-implantation development is probably not due to its conversion to NO but to other products such as polyamines [55], [58] or due to altered signaling pathways, for example, mTOR [59], [60].

### Conclusions

We report the first comprehensive study of the cell cycle during early phases of reprogramming after somatic cell nuclear transfer into the mouse oocyte. We conclude that the first cell division is entirely, and the second division partly controlled by maternal factors. At the four-cell stage, the delayed activation of essential embryonic cell cycle genes and the concomitant depletion of maternal cell cycle proteins may force blastomeres of cloned embryos to wait for replenishment of cell cycle molecules. Failing re-activation of these essential genes causes cloned cells to arrest, possibly explaining the high losses after nuclear transfer at this developmental stage. Non-systematic gene expression variances of fast and slow cleaving cloned embryos suggests that cell cycle genes and genes related to pluripotency and fetal formation are reprogrammed independently of each other, implying some stochastic component of reprogramming. The dys-regulation of the embryonic clock after somatic cell nuclear transfer does not cause an increase of M phase aberrancies. However, cloned embryos seem to be less tolerant to aneuploid cells at this developmental stage. We also report that an increased arginine supply facilitates blastocyst formation from cloned embryos.

**Figure 6:**
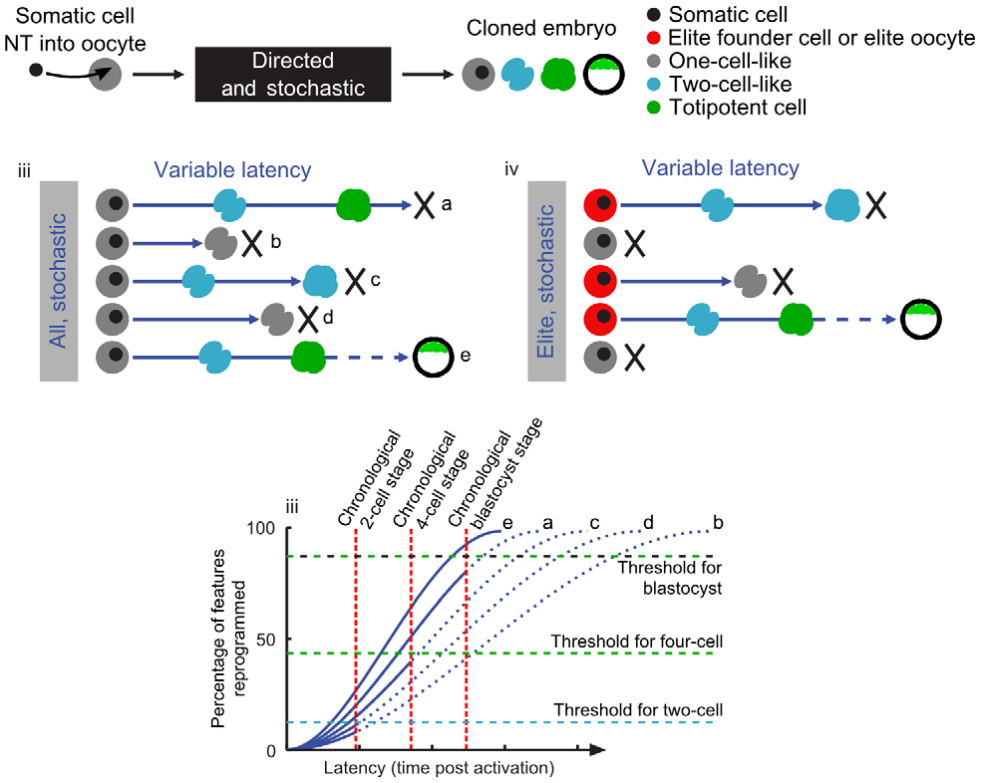
Proposed model of reprogramming mode, adapted from Hanna et al. [**8**]. After NT, reprogramming proceeds with directed and stochastic components (A) with variable latency to yield embryos of different developmental stages (B). Due to the stochastic component of the reprogramming process, some embryos have been reprogrammed more than others at a certain point in time (C). If a critical reprogramming threshold of genes essential for the respective chronologically timed embryonic stage is not reached, the embryo halts development (C). It cannot be excluded that certain oocytes reprogram better than others (elite oocytes, D), for example due to higher levels of certain factors of the “reprogrammome” [61].

In analogy to the proposed model of reprogramming in a scenario of induced pluripotency (iPSC) [8], our data suggests that reprogramming after somatic cell NT is a stochastic process with variable latency (Figure 6A,B). Reprogramming by the oocyte is orders of magnitude faster and more efficient than reprogramming by combination of transcription factors in iPSC derivation. This indicates that in oocytes the process also has an actively directed component constituted by, for example, remodeling complexes and chromatin modifying enzymes [8], [61], [62]. During iPSC formation on the other hand, the pluripotency factors need to wait until binding sites are exposed by chance (e.g., during replication). The embryo characteristics exert strong selective pressure on the reprogramming process: nuclei that did not reprogram a threshold of critical genes until a selective developmental time point on the embryonic clock do not have the chance to continue development (Figure 6C). It would be interesting to determine whether certain oocytes reprogram better than others (model of elite oocytes, Figure 6D), for example, through higher levels of certain transcription factors [63], enzymes, metabolites, mitochondria or the like.

## Materials and Methods

### Ethics Statement

This study was performed in strict accordance with the recommendations of the Federation of Laboratory Animal Science Associations (FELASA). The protocol was approved by the Landesamt für Natur, Umwelt und Verbraucherschutz (LANUV) of the state of North Rhine-Westphalia, Germany (Permit Number: G 9.93.2.10.36.07.254). Teratoma formation was approved by permit number 87-51-04-2010-A387. Mouse surgery was performed under Ketamine/Xylazine anesthesia, and every effort was made to minimize suffering.

### Mice

Six- to eight-week-old B6C3F1 (C57Bl/6J×C3H/HeN) mice were used as oocyte and cumulus cell donors. Mice expressing histone H_2b_-linked GFP made by C.T. and B.K. were a kind gift of Takashi Hiiragi. OG2 mice harboring an Oct4-GFP transgene [64] are available at the Jackson Laboratory (B6;CBA-Tg(Pou5f1-EGFP)2Mnn/J; stock number 004654). When generation of ESCs was intended, F1(129SV×OG2) cumulus cells were used as somatic donors for cloning, and OG2 sperm was used for intracytoplasmic sperm injection (ICSI). Mice were maintained in an environment with constant humidity, temperature and photoperiod (14L∶10D) to prevent reported seasonal differences of cleavages [65].

The histone H2b-GFP construct was made by cloning H2b-GFP fusion into pRN3 vector [66] to yield maximal stability of the transcripts and then further into pAct-tetR-pA (gift from M. Mallo), replacing the tetR with H2b-GFP. The H2b-GFP fragment together with the actin promoter was then inserted into p5′-Srycre-ins1+2 (gift from G. Scherer) between two insulators replacing 5′-Srycre, creating pAct-H2b-GFP-ins. pAct-H2b-GFP-ins was linearized and used for pronuclear injections into FVB/N pronuclear eggs according to standard techniques. Transgenesis of offspring was probed using PCR (primers 5′-AGT CCT GAG GGA TAC GTG C-3′ and 5′-GAC CAT GTG ATC TCT CTT TCC-3′).

### Somatic cell nuclear transfer (NT) and intra-cytoplasmic sperm injection (ICSI)

Micromanipulations were performed as previously described [40]. Fertilized control embryos were produced by ICSI, which underwent a similar amount of micromanipulation as embryos cloned by NT.

### In vitro culture of NT and ICSI embryos

In vitro culture of NT and ICSI embryos was performed as previously described [40]. α-MEM (containing amino acids) was purchased (Sigma cat. no. M4526). All media were supplemented with BSA (2 mg/mL) and gentamicin sulphate (50 µg/mL). For some experiments, α-MEM was supplemented with arginine (126 µg/mL) or with diethylenetriamine/nitric oxide adduct (DETA/NO; Sigma cat. no. D185; gift from S. Höing; 10 µM). Since the half-life of DETA/NO in the culture medium is short, a daily change with fresh-made medium was necessary.

### Embryo transfer in vivo

Embryos were transferred to oviducts of CD1 females that had been mated to vasectomized males three days ahead and plugged on the day of embryo transfer. This was performed 12 hours after scoring cleavage timing to three-cell stage, at 50 hpa.

### Time-lapse cinematography

Live fluorescence images of embryos expressing histone H_2b_-GFP and live bright-field images of wild-type embryos were captured with an UltraVIEW RS3 spinning disk confocal imaging system (PerkinElmer LAS, Jügesheim, Germany) fitted to an inverted microscope (TE 2000U, Nikon, Düsseldorf, Germany) using a 20× objective lens (N.A. 0.75). A field of view fitted approximately 12 embryos. The source was a 3-line (488, 568 and 647 nm) Argon/Krypton laser (Melles Griot). Optical sections were captured using a Hamamatsu ORCA ER digital camera. Embryos were placed in groups of 20 in a 20 µL drop of α-MEM on a 35 mm thin-bottom plastic dish (Greiner Bio-One, Lumox dish cat. no. 96077303), and overlaid with embryo-tested mineral oil (Sigma cat. no. M5310) previously washed with MilliQ water. A Tokai-Hit environmental mini-chamber maintained a gas phase of 5% CO_2_, 5% O_2_ and 90% N_2_ at 37°C. Multiple positions were accessed with a Ludl BioPoint 2 motorised stage (Chromaphor Analysen-Technik GmbH, Bottrop, Germany). Until 48 h post activation, bright-field images were captured every 20 min, using a 580/10 nm bandpass filter. After that until 96 h, sections were captured in fluorescence mode 5 µm apart, using 488 nm laser with 0.5 mW laser power at the lens and an exposure time of 1 s. These conditions allowed blastocyst formation of NT and ICSI embryos.

Time-lapse movies were evaluated to obtain the time of cleavage of the first 3 (bright-field) and the following 2 (fluorescence) cell cycles. For each cell of every embryo, the time between consecutive cleavages was determined and linked to development to the blastocyst stage. Cell cycle lengths obtained from 3 (1× arginine) respective 2 (2× arginine) independent experiments were averaged.

### Derivation of ESCs

Derivation of ESC lines from NT blastocysts was performed as described previously [67]. After zona pellucida removal with Tyrode's solution (Sigma), blastocysts were transferred onto a feeder layer of irradiation-inactivated mouse embryonic fibroblasts in 48-well dishes. At day 7 after NT, blastocysts attached to the feeders forming trophoblastic outgrowths. The inner cell masses (ICMs) were removed with a polished glass pipette, digested with 0.25% trypsin-EDTA (Invitrogen GmbH, Darmstadt, Germany) and reseeded onto fresh feeder cells on a 96-well plate. Passage zero ESCs (after initial plating of the dissociated ICMs) were grown for 5 days in culture and further expanded by subsequent passaging (2–3 days) onto larger well sizes. ESC culture media consisted of knockout DMEM (Gibco) with 15% serum replacement (Invitrogen), 5% fetal bovine serum (FBS; BioWest, Nuaillé, France), glutamine and penicillin/streptomycin (Invitrogen), nonessential amino acids (PAA Laboratories, Pasching, Austria), mercaptoethanol (Invitrogen), 2000 Units·mL^−1^ leukemia inhibitory factor (produced in house) and 50 µM MEK1 inhibitor PD98059 (Cell Signaling Technology, Inc., Danvers, MA, U.S.A.).

### Teratoma formation by ESCs

Teratoma formation was assessed as described [67]. Single cell-suspended ESCs were injected subcutaneously in the neck region of scid (NOD.CB17-*Prkdc^scid^*/J) male mice (1–2·10^6^ cells per 200 µL). Mice were sacrificed 3 weeks after injection; teratomas were excised, fixed in 4% PFA-PBS for 48 h. Successive washes in running water, a gradient of 40%, 70%, 90% and 100% ethanol, 100% isopropanol and 100% xylol, were performed, 30 min each wash. Teratomas were embedded in paraffin, stained with hematoxylin and eosin, and sectioned for histological examination.

### In vitro differentiation of ESCs

Differentiation ability of the derived NT-ESCs and control ESCs in vitro was performed as described [67]. Embryoid bodies (EBs) were produced using the hanging drop method (500 ESCs per 20 µl), after removal of feeder cells by sedimentation. After 3 days in culture with ESC media, EBs were plated on gelatinized plates for endoderm and mesoderm differentiation, and on matrigel-coated plates for ectoderm differentiation. For mesoderm and endoderm differentiation, EBs were cultured in ESC media containing 15% and 20% FBS, respectively, without leukemia inhibitory factor. For ectoderm differentiation, EBs were cultured in media consisting of a 1∶1 mixture of DMEM/F12 (1∶1; Gibco) and Neurobasal medium (Gibco), 0.2% BSA fraction V (7.5%; Gibco), 0.5% N2 supplement (Invitrogen, Darmstadt, Germany), 1% B27 supplement (Invitrogen), glutamine/penicillin/streptomycin, mercaptoethanol and 20 µM activin-like kinase inhibitor SB431542 (Ascent Scientific, Bristol, UK). After two weeks of differentiation, cells were fixed in 4% PFA in PBS for 10 min and rinsed with PBS. Residual PFA was quenched using 50 mM glycine in PBS. Fixed cells were permeabilized with 0.2% Triton X-100 in PBS for 10 min, rinsed with PBS and blocked with 2% filtered FBS in PBS-T for 45 min. Primary antibodies for ectoderm (Tuj1 beta-III-tubulin, Sigma T8660, 1∶1000) and for endoderm (Sox17, R&D AF1924, 1∶50) diluted in blocking solution were applied and incubated overnight at 4°C. After washing with PBS-T, alexa-fluor-568 coupled secondary antibody (Invitrogen, anti-goat A11079 and anti-mouse A11061) was applied in a 1∶500 dilution in PBS/20% blocking solution, incubated for 1 h and then washed with PBS-T. Hoechst-33342 (Fluka) 5 µg·mL^−1^ in PBS was used for DNA counterstaining. Finally, samples were analyzed for fluorescence.

### Illumina bead chip hybridizations

Embryos were scored *fast*, *intermediate* or *slow* according to the time spent until three-cell stage was reached. Embryos of the *fast* and the *slow* group that had reached eight-cell stage on the next day were lysed and frozen at −80°C until processing. We performed two independent biological replicates. Total RNA was isolated using the MicroRNeasy Kit (Qiagen, Hilden, Germany). In order to generate enough RNA for the subsequent microarray analysis, a two-round linear amplification protocol employing a linear amplification kit (TargetAmp aRNA amplification kit, Epicentre Biotechnologies, Madison, WI, U.S.A.) was adopted to generate biotin-labelled cRNA, 1.5 µg of which was used for each hybridization reaction. CRNA samples were hybridised onto Illumina mouse 8 BeadChips. Washing, Cy3-streptavidin staining, and scanning were performed on the Illumina iScan (Illumina, San Diego, CA, U.S.A.) platform using reagents and following protocols supplied by the manufacturer. MIAME compliant data was made accessible in the public microarray repository ArrayExpress (http://www.ebi.ac.uk/arrayexpress) under accession number E-TABM-1182.

All expression data analysis was carried out using Bioconductor [68], an open source project based on the R programming language [69]. Un-normalised bead-summary data (including regular probe profile and control probe profile), with associated probe annotation, were output from Illumina GenomeStudio. Raw data were background-subtracted, transformed for variance stabilization (VST) and normalised using robust splines (RSN) using lumi R package [70]. Normalised data were filtered for significant expression on the basis of negative control beads. Fold changes and standard errors were estimated by fitting a linear model for each gene using limma (linear models for microarray) R package [71]. Mapping was performed using lumiMouseAll.db R package [72].

### Selection of maternal and embryonic transcripts

We used published data sets [35], [36], [37] to select transcripts specific to oocytes/1-cell embryos (maternal) or four-cell to blastocyst stage embryos (embryonic). The data set from Hamatani et al (GSE936) was downloaded from gene expression omnibus (http://www.ncbi.nlm.nih.gov/geo) using GEOquery [73], flag filtered and quantile normalised. The data set from Wang et al (E-MEXP-51) was downloaded from ArrayExpress (http://www.ebi.ac.uk/arrayexpress), cubic splines quantile normalised and MAS 5.0 background corrected using affy [74]. The data set from Zheng et al (GSE1749) was downloaded from Gene Expression Omnibus, calibrated and variance stabilised using vsn [75]. Fold changes and standard errors were estimated for data sets individually by fitting a linear model for each gene using limma [71]. Genes specific to either oocytes/1-cell embryos or four-cell to blastocyst stage embryos in one or more of the data sets were then designated *maternal* or *embryonic*, respectively.

### Amino acid analysis

Embryos were cultured in groups of 20 (one-cell) down to 5 (morula/blastocyst) for 16.5 to 18 h in pre-equilibrated 5 µL drops of KSOM medium supplemented with 0.4% BSA, 0.2 mM L-glutamine and amino acids [76], under oil. The spent medium was stored at −80°C. Following thawing, a 2 µL aliquot was removed from each spent drop and diluted 1∶12.5 in HPLC grade water. Embryo-free control drops were incubated alongside the embryo containing drops to allow for any non-specific amino acid degradation or appearance.

Analysis of amino acid content was adapted from the method of Houghton et al. [49]. Briefly, reverse-phase HPLC was performed using an Agilent 1100 HPLC with fluorescence detector and a 50×4.6 mm Gemini column (Phenomenex, Macclesfield, Cheshire, UK). Derivatisation was achieved by reaction of 10 µL sample with an equal volume of *o*-phthaldialdehyde containing 0.2% 2-mercaptoethanol. The elution gradient operated at a flow rate of 2.5 mL/min. Solvent A consisted of 5 mL tetrahydrofuran (Fisher Chemicals, Loughborough, Leicestershire, UK), 200 mL methanol, and 800 mL sodium acetate (83 mmol/L, pH 5.9). Solvent B contained 800 mL methanol and 200 mL sodium acetate (83 mmol/L, pH 5.9). Using this method it was not possible to detect proline and cysteine.

Results are expressed as amino acid depletion/appearance in pmol·embryo^−1^·h^−1^ ± SEM. The term *turnover* describes the sum of amino acid depletion and appearance. Results of 3 to 13 single embryos were summarised. R package gplots was used to draw the heatmap. Distance of amino acids was calculated as 1 – Spearman correlation coefficient; distance measure of groups of embryos is Euclidean distance. Clustering of amino acids was performed using complete linkage; clustering of groups of embryos was performed using Ward's minimum variance method.

### Statistical analysis

All statistical analysis was performed in R [69].

## Supporting Information

Movie S1 **High light vulnerability of cloned embryos.** Our initial bright field time-lapse cinematography attempts of NT embryos resulted in two-cell stage arrest, while ICSI embryos (14 embryos top left) formed blastocysts.
(MOV)

Movie S2 **Time-lapse movie of representative ICSI embryos from 10:28 hours post activation (hpa) until 96∶32 hpa.** Until 45∶48 hpa, images were taken in bright field, after that stacks of H_2b_-GFP fluorescence images were recorded. Of nine embryos, five carried the H_2b_-GFP allele, and five formed blastocysts.
(MOV)

Movie S3 **Time-lapse movie of representative NT embryos from 10:28 hours post activation (hpa) until 96∶32 hpa.** Until 45∶48 hpa, images were taken in bright field, after that stacks of H_2b_-GFP fluorescence images were recorded. Two of six embryos formed blastocysts.
(MOV)

Movie S4 **Example of cytokinesis failure.** At the two- to four-cell stage transition, the bottom nucleus divides, but cytokinesis fails. At the four- to eight-cell transition, the two nuclei fuse.
(MOV)

Movie S5 **Example of cell fusion.** At the four-cell stage, the bottom two nuclei, which are not sister nuclei, fuse. The fused cell then divides and gives rise to tetraploid daughter cells. The embryo forms a blastocyst.
(MOV)

Movie S6 **ESCs derived from fast NT embryos differentiated into beating cardiomyocytes.**
(MP4)

Movie S7 **ESCs derived from slow NT embryos differentiated into beating cardiomyocytes.**
(MP4)

Movie S8 **ESCs derived from fertilized embryos differentiated into beating cardiomyocytes.**
(MP4)

Figure S1 **Accuracy of predicting developmental success from combining two measured variables of cleavage timing in cloned (NT) and fertilized (ICSI) embryos up to the 4-cell stage (** ***F*** **-score).** *F*, *sn* (sensitivity), and *pr* (precision) in the title relate to the overlap of the grey and the orange highlighted window. Green dots, embryo developed to blastocyst stage; red dots, embryo did not develop to blastocyst stage. Also combination of two parameters does not allow prediction of blastocyst formation of NT embryos with *F*>0.49.
(PDF)

Figure S2 **Accuracy of predicting developmental success from combining three measured variables of cleavage timing in cloned (NT) and fertilized (ICSI) embryos up to the 4-cell stage (** ***F*** **-score).** *F*, *sn* (sensitivity), and *pr* (precision) in the title relate to the overlap of the grey and the orange highlighted window. Green dots, embryo developed to blastocyst stage; red dots, embryo did not develop to blastocyst stage. Also combination of three parameters does not allow prediction of blastocyst formation of NT embryos with *F*>0.49.
(PDF)

Figure S3 **Correlation of cell cycle lengths of daughter cells to mother cell.** ICSI embryos were consistent in their cleavage pace, that is, a blastomere that cleaved early was likely to cleave early in the next cell cycle. NT embryos only maintained their cleavage speed after the eight-cell stage, while second and third cell cycles were negatively correlated. Green, embryo developed to blastocyst stage; red, embryo did not develop to blastocyst stage. *r^2^*, Pearson correlation coefficient with *p*, p-value of that correlation coefficient is significantly different from zero.
(PDF)

Figure S4 **Correlation of cell cycle lengths of sister cells.** The duration of the cell cycle for one blastomere and its sister blastomere always correlated. Green, embryo developed to blastocyst stage; red, embryo did not develop to blastocyst stage. *r^2^*, Pearson correlation coefficient with *p*, p-value of that correlation coefficient is significantly different from zero.
(PDF)

Figure S5 **In vitro differentiation of ESCs derived from fast and slow NT.** NT-ESCs from fast and slow embryos as well as control ESCs derived from fertilized embryos (FD) were differentiated into ectoderm (A) and endoderm (B) using in vitro protocols. Successful differentiation was visualized using immunocytochemistry (antibody, red) for neuron-specific class III β-tubulin (Tuj1; ectoderm) and Sox17 (endoderm). Nuclei were counterstained with Hoechst (blue). Mesoderm formation was confirmed by the appearance of beating cardiomyocytes in culture (movies S6, S7, S8).
(TIF)

Table S1 **Light exposure affects cloned embryo viability.** Although an optimized combined bright field and fluorescence time-lapse cinematography protocol improved survival, light exposed cloned embryos developed to blastocyst with significantly lower frequency than non-imaged controls. For fertilized control embryos, the difference in development of imaged and non-imaged was not significant. The *p*-value of Fisher's exact test is shown in bottom row.
(DOC)

Table S2 **Development to blastocyst stage after aberrant M phase event at the four-/eight-cell transition.** Time-lapse records were analyzed for errors in cell divisions leading to aneuploid daughter cells in the third cleavage division, and development to blastocyst stage was tracked. n, number of embryos with at least one or without aberrant M phase at the four-/eight-cell transition, which developed or did not develop to blastocyst stage.
(DOC)
