# Supplementary figures and images for: Nuclear Reprogramming: Kinetics of Cell Cycle and Metabolic Progression as Determinants of Success
Source: PLoS One. 2012 Apr 18;7(4):e35322. doi: 10.1371/journal.pone.0035322 (PMC3329427; doi:10.1371/journal.pone.0035322)

NT embryos;  $F = 0.488$ ,  $sn = 0.909$ ,  $pr = 0.333$

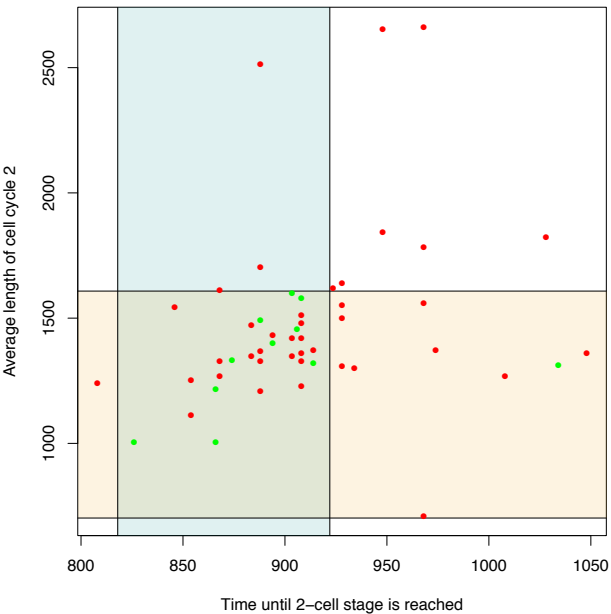

ICSI embryos;  $F = 0.778$ ,  $sn = 0.778$ ,  $pr = 0.778$

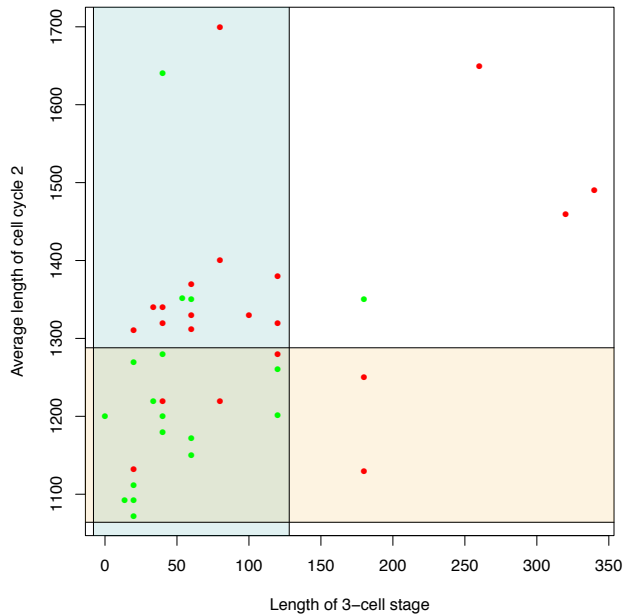

Supplement: Figure S1 — Accuracy of predicting developmental success from combining two measured variables of cleavage timing in cloned (NT) and fertilized (ICSI) embryos up to the 4-cell stage ( F -score). F, sn (sensitivity), and pr (precision) in the title relate to the overlap of the grey and the orange highlighted window. Green dots, embryo developed to blastocyst stage; red dots, embryo did not develop to blastocyst stage. Also combination of two parameters does not allow prediction of blastocyst formation of NT embryos with F>0.49. (PDF) [file pone.0035322.s009.pdf]

ICSI embryos;  $F = 0.778$ ,  $sn = 0.778$ ,  $pr = 0.778$

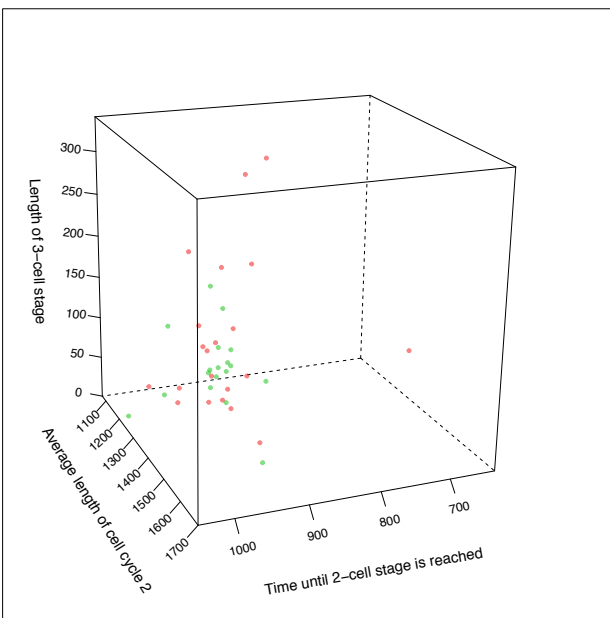

ICSI embryos;  $F = 0.778$ ,  $sn = 0.778$ ,  $pr = 0.778$

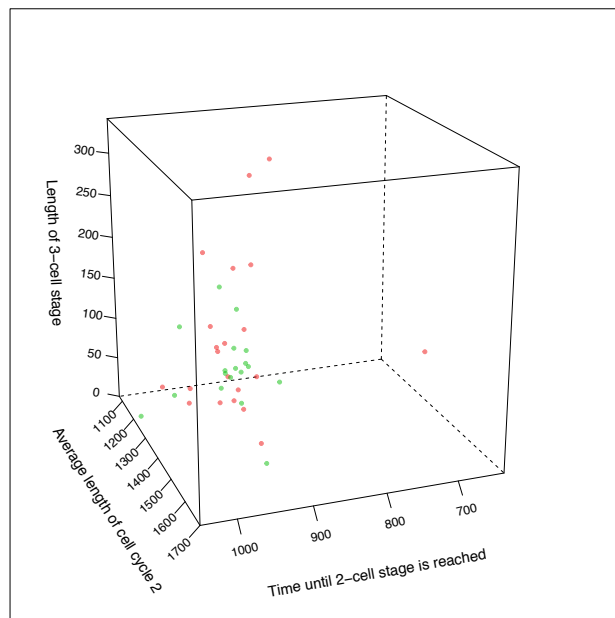

NT embryos;  $F = 0.488$ ,  $sn = 0.909$ ,  $pr = 0.333$

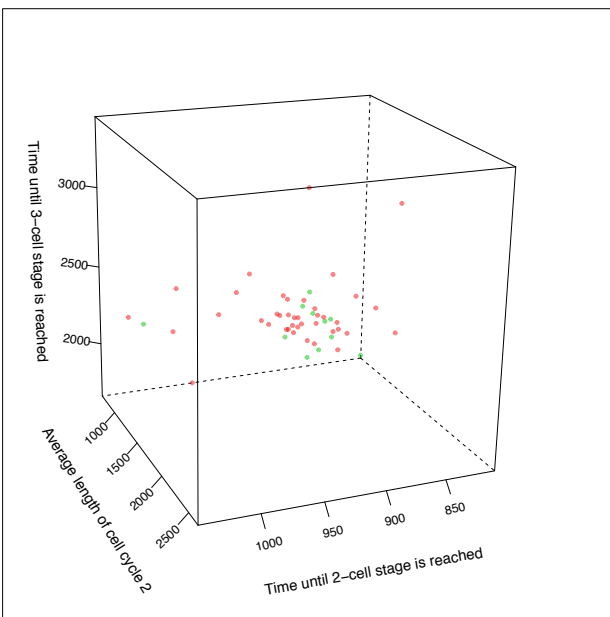

NT embryos;  $F = 0.488$ ,  $sn = 0.909$ ,  $pr = 0.333$

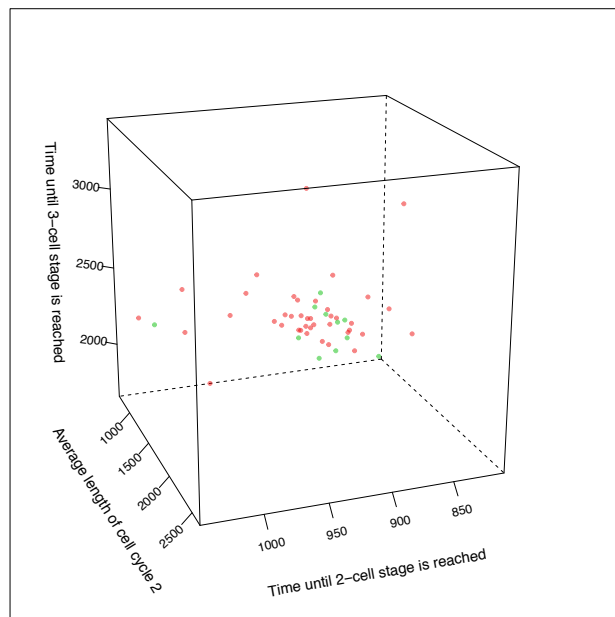

Supplement: Figure S2 — Accuracy of predicting developmental success from combining three measured variables of cleavage timing in cloned (NT) and fertilized (ICSI) embryos up to the 4-cell stage ( F -score). F, sn (sensitivity), and pr (precision) in the title relate to the overlap of the grey and the orange highlighted window. Green dots, embryo developed to blastocyst stage; red dots, embryo did not develop to blastocyst stage. Also combination of three parameters does not allow prediction of blastocyst formation of NT embryos with F>0.49. (PDF) [file pone.0035322.s010.pdf]

# ICSI

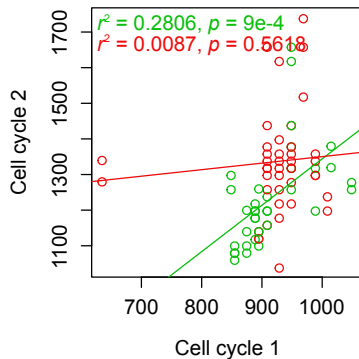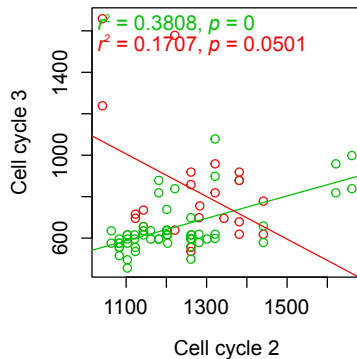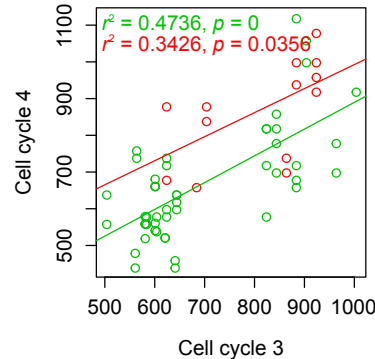

# NT

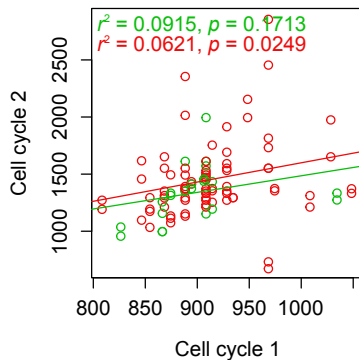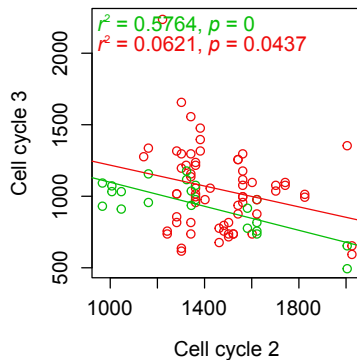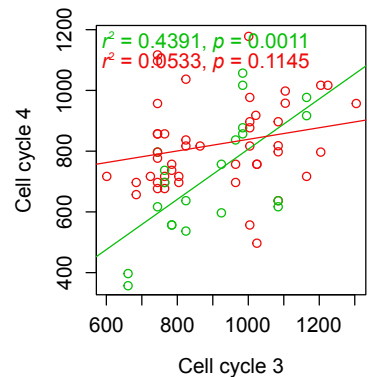

Supplement: Figure S3 — Correlation of cell cycle lengths of daughter cells to mother cell. ICSI embryos were consistent in their cleavage pace, that is, a blastomere that cleaved early was likely to cleave early in the next cell cycle. NT embryos only maintained their cleavage speed after the eight-cell stage, while second and third cell cycles were negatively correlated. Green, embryo developed to blastocyst stage; red, embryo did not develop to blastocyst stage. r2, Pearson correlation coefficient with p, p-value of that correlation coefficient is significantly different from zero. (PDF) [file pone.0035322.s011.pdf]

# ICSI

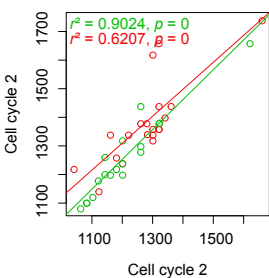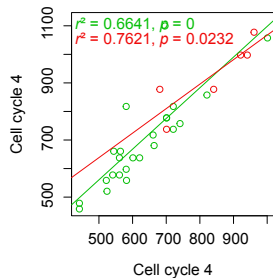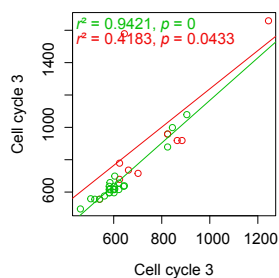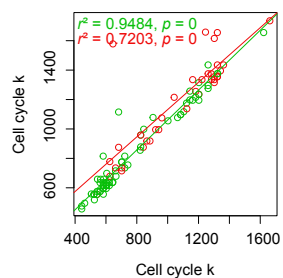

# NT

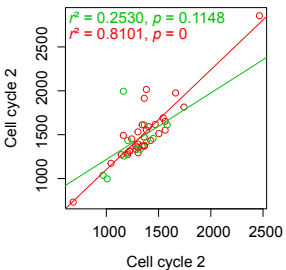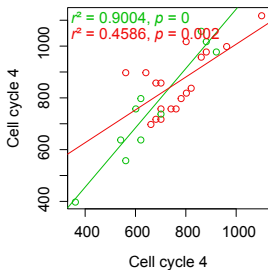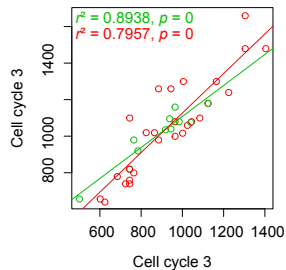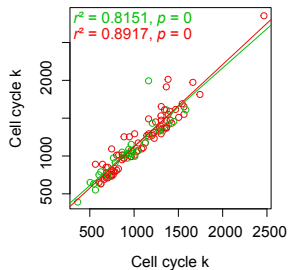

Supplement: Figure S4 — Correlation of cell cycle lengths of sister cells. The duration of the cell cycle for one blastomere and its sister blastomere always correlated. Green, embryo developed to blastocyst stage; red, embryo did not develop to blastocyst stage. r2, Pearson correlation coefficient with p, p-value of that correlation coefficient is significantly different from zero. (PDF) [file pone.0035322.s012.pdf]

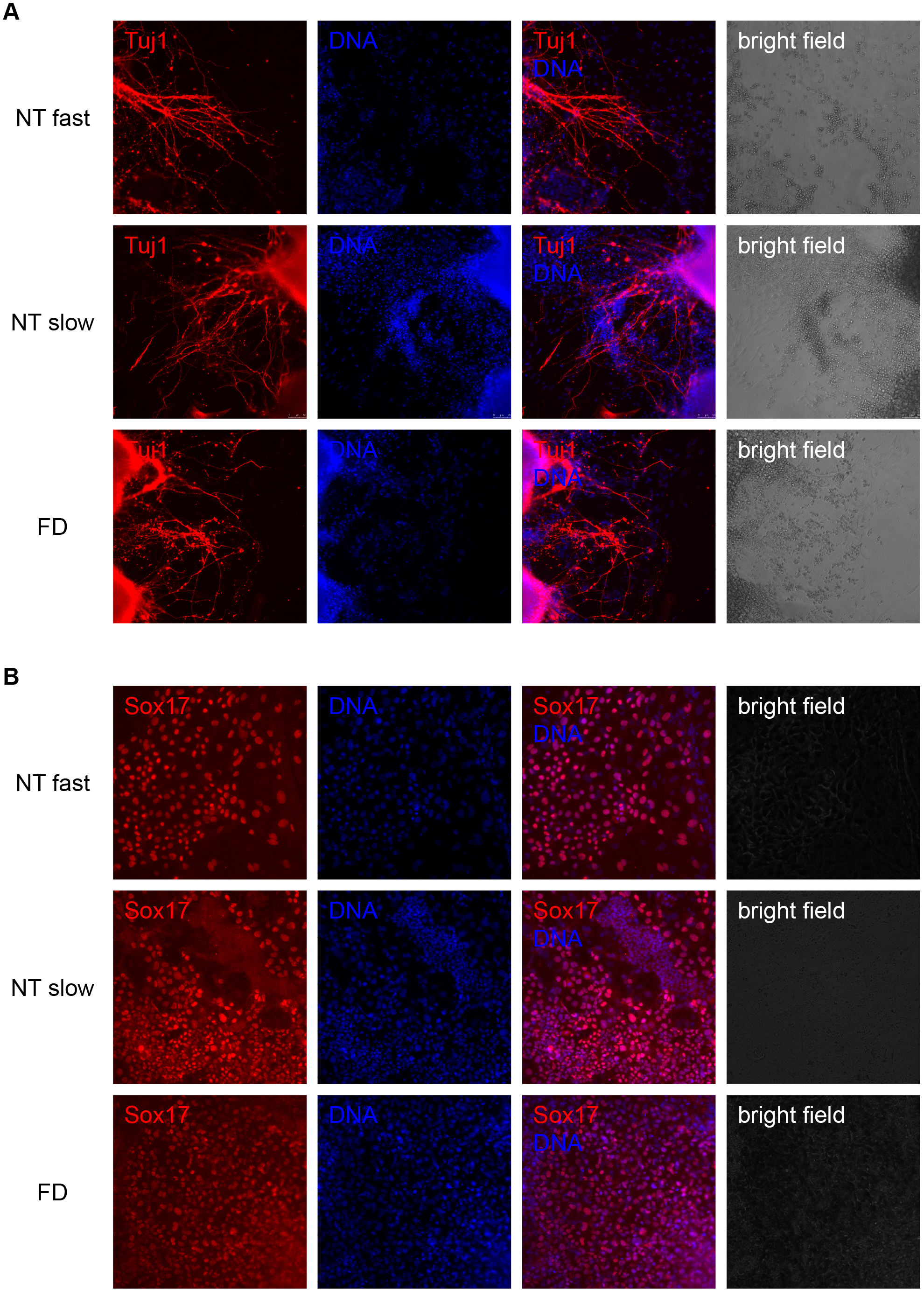

Supplement: Figure S5 — In vitro differentiation of ESCs derived from fast and slow NT. NT-ESCs from fast and slow embryos as well as control ESCs derived from fertilized embryos (FD) were differentiated into ectoderm (A) and endoderm (B) using in vitro protocols. Successful differentiation was visualized using immunocytochemistry (antibody, red) for neuron-specific class III β-tubulin (Tuj1; ectoderm) and Sox17 (endoderm). Nuclei were counterstained with Hoechst (blue). Mesoderm formation was confirmed by the appearance of beating cardiomyocytes in culture (movies S6, S7, S8). (TIF) [file pone.0035322.s013.tif]
