# Supplementary material for: Nuclear Reprogramming: Kinetics of Cell Cycle and Metabolic Progression as Determinants of Success
Source: PLoS One. 2012 Apr 18;7(4):e35322. doi: 10.1371/journal.pone.0035322 (PMC3329427; doi:10.1371/journal.pone.0035322)
Supplement: Table S1 — Light exposure affects cloned embryo viability. Although an optimized combined bright field and fluorescence time-lapse cinematography protocol improved survival, light exposed cloned embryos developed to blastocyst with significantly lower frequency than non-imaged controls. For fertilized control embryos, the difference in development of imaged and non-imaged was not significant. The p-value of Fisher's exact test is shown in bottom row. (DOC) [file pone.0035322.s014.doc]

| % blastocysts (*n* one-cells) | NT | ICSI |
| --- | --- | --- |
| imaged | 13.4 (82) | 40.0 (45) |
| not imaged | 33.9 (165) | 44.2 (165) |
| p | 6.8·10^‑3^ | 0.735 |
