# Supplementary material for: Nuclear Reprogramming: Kinetics of Cell Cycle and Metabolic Progression as Determinants of Success
Source: PLoS One. 2012 Apr 18;7(4):e35322. doi: 10.1371/journal.pone.0035322 (PMC3329427; doi:10.1371/journal.pone.0035322)
Supplement: Table S2 — Development to blastocyst stage after aberrant M phase event at the four-/eight-cell transition. Time-lapse records were analyzed for errors in cell divisions leading to aneuploid daughter cells in the third cleavage division, and development to blastocyst stage was tracked. n, number of embryos with at least one or without aberrant M phase at the four-/eight-cell transition, which developed or did not develop to blastocyst stage. (DOC) [file pone.0035322.s015.doc]

| ICSI | Embryos with aberrant M phase | Embryos without aberrant M phase | NT | Embryos with aberrant M phase | Embryos without aberrant M phase |
| --- | --- | --- | --- | --- | --- |
| Development to blastocyst | 13 | 2 |  | 7 | 0 |
| No development to blastocyst | 7 | 1 |  | 38 | 9 |
